# The Interplay Between Viral-Derived miRNAs and Host Immunity During Infection

**DOI:** 10.3389/fimmu.2019.03079

**Published:** 2020-01-23

**Authors:** Richa Mishra, Ashish Kumar, Harshad Ingle, Himanshu Kumar

**Affiliations:** ^1^Laboratory of Immunology and Infectious Disease Biology, Department of Biological Sciences, Indian Institute of Science Education and Research, Bhopal, India; ^2^Department of Dermatology, School of Medicine, University of California, Davis, Sacramento, CA, United States; ^3^Department of Medicine, Washington University School of Medicine, Saint Louis, MO, United States; ^4^Laboratory of Host Defense, WPI Immunology, Frontier Research Centre, Osaka University, Osaka, Japan

**Keywords:** host immunity, viruses, MicroRNAs, infection, pathogenesis

## Abstract

MicroRNAs are short non-coding RNAs that play a crucial role in the regulation of gene expression during cellular processes. The host-encoded miRNAs are known to modulate the antiviral defense during viral infection. In the last decade, multiple DNA and RNA viruses have been shown to produce miRNAs known as viral miRNAs (v-miRNAs) so as to evade the host immune response. In this review, we highlight the origin and biogenesis of viral miRNAs during the viral lifecycle. We also explore the role of viral miRNAs in immune evasion and hence in maintaining chronic infection and disease. Finally, we offer insights into the underexplored role of viral miRNAs as potential targets for developing therapeutics for treating complex viral diseases.

## Introduction

Until the twenty first century, it was assumed that more than 95% of the eukaryotic genome is “junk” DNA; however, the advent of next-generation sequencing and high throughput functional screening has highlighted the regulatory functions of the non-coding genome. Around 50–85% of the mammalian genome is transcribed, with at least some non-coding RNA transcript, which includes microRNA (miRNA), siRNA, piwi-interacting RNA (piRNA), long non-coding RNA (lncRNA), and circular RNA (circRNA) (1–4). In recent years, the role of miRNAs in the development of immune responses in viral infections has been a subject of immense research interest.

MicroRNAs are small RNA molecules (18–22 nt) that play a crucial role in the regulation of gene expression by binding to the 3′ untranslated region (3′UTR) of target messenger RNAs (mRNAs) (5–7). miRNA-mRNA interaction results in mRNA degradation or translation inhibition, thereby resulting in reduced gene expression, thereby modulating the biological function (8, 9). Various studies indicate that miRNA can be derived from an intronic region of coding and non-coding genes, an exonic region of non-coding genes, and intragenic regions (10, 11). In addition to the hundreds of eukaryotic cellular miRNAs, miRNAs of viral origin (also known as v-miRNAs) have been discovered that can function as post-transcriptional gene regulators to host as well as viral genes (12, 13).

In this review, we summarize the miRNA-like non-coding RNAs encoded by DNA and RNA viruses and their roles in the evasion of host immunity. Finally, we discuss the possible role of these v-miRNAs as potential targets for developing therapeutics for the treatment of viral diseases.

## Discovery and Origin of Viral miRNAs

Two different approaches have been used to identify v-miRNAs. (i) The use of computational tools to predict the secondary structure of the precursor of v-miRNAs (pre-v-miRNAs). This approach usually results in a large number of false positives; however, it can lead to the identification of less abundant miRNA (14, 15). (ii) Sequencing of cloned small RNA molecules; however, less abundant miRNA may be left out (15–18). The first v-miRNAs were identified in 2004 in the Epstein-Barr virus (EBV) by cloning the small RNAs from Burkitt's lymphoma cell line latently infected with EBV. Analysis of the genomic region flanking the small RNA molecules suggested characteristic miRNA gene-like structures (19). The EBV-miRNAs miR-BHRF1-1, miR-BHRF1-2 and miR-BHRF1-3 and miR-BART1 and miR-BART2 originated from two regions in BHRF1 and BART mRNAs (19). To date, EBV is known to encode 44 v-miRNAs from 25 double-stranded RNA precursors (20). Work by Pfeffer et al. suggested that other DNA viruses might also express miRNAs to modulate host and viral gene expression (19). Notably, more than 250 v-miRNAs have been identified, and the majority of them are accounted for in the DNA viruses of the herpesvirus family (12, 21).

**Figure 1:**
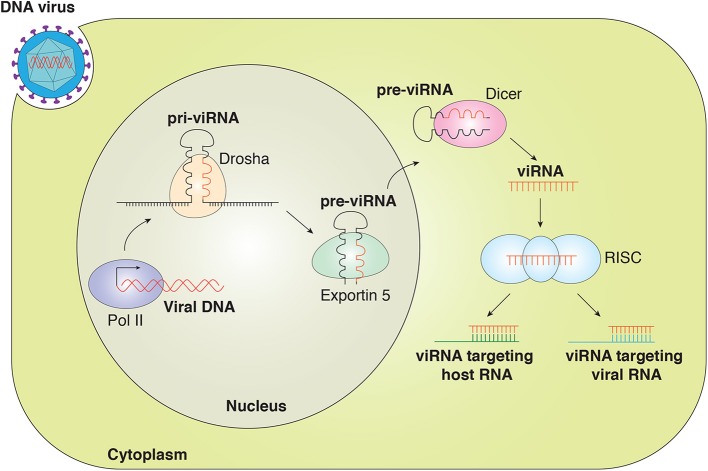
Canonical viral miRNAs are transcribed as pri-v-miRNAs from the genome of DNA viruses in the nucleus. Cleavage of pri-v-miRNAs by Drosha results in pre-v-miRNA that is exported to the cytoplasm via Exportin 5. In the cytoplasm, pre-v-miRNAs undergo cleavage by Dicer to generate the mature v-miRNAs that are loaded in the multiprotein RISC complex to target the host and viral mRNA transcript. v-miRNAs (viRNA).

Studies have indicated that viruses utilize the cellular machinery to encode miRNA. Similarly to eukaryotic miRNAs, v-miRNAs are generated by the Drosha and Dicer machinery (5, 22). The viral miRNA gene is transcribed by RNA polymerase II (RNA pol II) or Pol III to generate primary miRNA (pri-miRNA) that is then processed by a complex of Drosha/DiGeorge syndrome (DGCR8) within the nucleus to generate ~70-nucleotide (nt) pre-miRNA. The pre-miRNA is exported out of the nucleus by exportin-5, where pre-miRNA is further processed by endonuclease Dicer to yield mature miRNA duplex (21–25 nt). One strand of the duplex is loaded into the RNA-induced silencing complex (RISC) containing Argonaute 2 (Ago2). The miRNA-RISC complex interacts with the target transcript and inhibits gene expression (Figure 1) (23–26).

## v-miRNAs in DNA Viruses

### γ-Herpesvirus-Encoded v-miRNAs

EBV is a γ-herpesvirus that infects 90% of the world population and is associated with various epithelial and lymphoid malignancies including Burkitt's lymphoma (BL), gastric carcinoma (GC), Hodgkin's lymphoma (HL), and nasopharyngeal carcinoma (NPC) (27–30). In infected cells, EBV can establish two phases of infection known as latent and lytic. EBV primarily infects the human oropharynx epithelial cells, followed by replication and spread to B cells. This results in latent infection in B cells, epithelial cells, and natural killer/T cells (31). Latent EBV infection is a substantial cause of many human malignancies (32). Under certain conditions, EBV switches from latent to lytic phase, a stage where it expresses a repertoire of more than 80 genes, accompanied by viral DNA replication and finally leading to the production of progeny virus particles (33, 34). The majority of the EBV miRNAs are transcribed from the BART and BHRF1 regions. BART and BHRF1 transcripts encode 40 and 4 mature miRNAs, respectively (34–36). BART and BHRF1 transcripts express different miRNAs during different phases of EBV latency (37). EBV-encoded BART transcripts are detected in EBV-associated NPC biopsy or EBV-positive cell lines *in vitro* (38, 39).

Following the identification of EBV-encoded miRNA, several reports identified miRNA expressed by Kaposi's sarcoma-associated herpesvirus (KSHV/HHV8), another member of the γ-herpesvirus family. KSHV is known to cause Kaposi's sarcoma (KS), primary effusion lymphoma (PEL), Multicentric Castleman's disease (MCD), and KSHV inflammatory cytokine syndrome (40–42). A total of 25 mature miRNA processed from 12 different pre-miRNAs have been identified in KSHV and are in the latency-associated region (43, 44). Out of the 12 pre-miRNAs, 10 pre-miRNAs are located between the kaposin and open reading frame 71 (ORF71) genes, while miR-K10 is located within the kaposin gene, and miR-K12 is mapped to the 3′-UTR of kaposin. Although all known KSHV v-miRNAs are expressed during the viral-latent phase, v-miRNAs originating from pre-miR-K10 and pre-miR-K12 are further enhanced during the viral-lytic phase (15, 16, 44, 45). Also, several of the KSHV-encoded v-miRNAs share the seed sequence with human-encoded miRNAs and therefore regulate many target genes. KSHV miRNAs help in maintaining KSHV latency and interfere with the host immune system by regulating viral and cellular gene expression, ultimately contributing to KS development (46).

### β-Herpesvirus-Encoded v-miRNAs

Like γ-herpesvirus, α- and β-herpesvirus are also found to express v-miRNAs. The β-herpesvirus human cytomegalovirus (HCMV) is commonly found in the human population and has the largest genome, 230 kb of double-stranded DNA (dsDNA), among the herpesvirus family (47). HCMV causes serious life-threatening diseases in patients with a compromised immune system such as the human immunodeficiency virus (HIV) infection or patients undergoing immunosuppressive therapies (48, 49). v-miRNAs encoded by HCMV were first identified in 2005 (16). The study predicted and cloned nine pre-miRNAs, which were later validated in two independent studies by Northern blotting (50, 51). More studies on HCMV miRNAs identified additional miRNAs using deep sequencing technology (ref). A total of 26 HCMV v-miRNAs have been identified to date, dispersed throughout the genome (16, 51, 52). The HCMV miRNAs target multiple host genes involved in immune response and cell cycle control and thereby enhance HCMV virulence (53, 54).

HCMV miRs, namely, miR-UL-112-1, US25-1, US25-2, US25-2-5p, US5-1, US33-5p, and ULD148D, have been shown to inhibit HCMV DNA viral replication by targeting multiple host and viral regulatory genes (discussed in the following sections) (55–63). miR-UL112, US25-2-3p, and US4-1 modulate immune recognition by cytotoxic T lymphocyte (CTL) and natural killer (NK) cells (64–67). Similarly, the HCMV miRs UL112-3p, US5-1, UL112-1, US25-1-5p, and UL148D target multiple host inflammatory genes and result in reduced inflammatory response (53, 54, 68–71). Also, UL148D and UL36-5p are found to inhibit programmed cell death by repressing the expression of cellular genes involved in the regulation of apoptosis (72–74). Altogether, HCMV miRNAs play an important role in regulating the expression of host and viral genes to induce latent infection.

### α-Herpesvirus-Encoded v-miRNAs

Herpes simplex virus (HSV) has two members, HSV-1 and HSV-2, which are known to cause oral or genital herpes lesions (75). v-miRNAs in HSV were first identified in 2006, and, to date, HSV-1 and HSV-2 are known to encode 27 and 24 functional v-miRNAs, respectively (76–78). Few of the HSV-1 and HSV-2 v-miRNAs share the same seed sequence (77, 78). Like γ-herpesvirus, HSV-1 and HSV-2 v-miRNAs are associated with latency-associated transcript and are expressed during the latent phase of infection (79).

Almost all herpesviruses encode their own v-miRNAs except varicella-zoster virus (VZV). Many small-RNA sequencing studies have been performed for VZV, but VZV v-miRNAs have not yet been identified (80).

### Papillomavirus (PV)

Human papillomaviruses (HPV) preferentially infect the keratinocytes of mucous membranes or skin and cause numerous benign and malignant lesions at different anatomical locations. HPV infection is a common cause of cervical cancer (81–84). HPV infection is associated with varying proportions of other cancers of the anogenital tract, head and neck region, and skin (85).

The first report for the prediction of HPV-encoded miRNAs came in 2011. In that study, the authors predicted the HPV-encoded miRNAs in several HPV types. They predicted the pre-miRNAs using a computational algorithm and compared the conserved mature miRNAs with currently known miRNAs. Predicted HPV miRNAs related to miR-466,-467, and -669 were common and specific to the mucosal HPV types. Also, the authors observed that HPV-38 expresses a miRNA conserved to human let-7a (86). In another study, the authors generated small RNA libraries of 10 HPV-associated cervical cancer and two HPV16-harboring cell lines. From the sequencing data, nine putative HPV miRNAs were discovered. Four HPV-encoded miRNAs (two by HPV16, one by HPV38, and one by HPV68) were validated (87).

Similarly, another study developed miRNA discovery by using a forced genome expression (miDGE) tool for the identification of miRNAs. The study screened 73 different PV genomes using miDGE and observed that most of the PV genomes are unlikely to encode viral miRNAs. However, they could identify and validate five different miRNAs (hpv17-miR-H1, hpv37-miRH1, hpv41-miR-H1, fcpv1-miR-F1, and fcpv1-miR-F2 encoded by four different PVs: HPV17, 37, 41, and FcPV1). These HPV miRNAs targeted transcripts corresponding to the early region of the HPV genome (18).

### Hepadnavirus

Hepatitis B virus (HBV) is the best-known member of the *Hepadna* virus family. Infection with HBV can cause chronic hepatitis, liver cirrhosis, and hepatocellular carcinoma (HCC). HBV has infected 2 billion people worldwide (88). In comparison to healthy individuals, HBV-infected patients have a 100-fold higher risk of development of HCC (89).

To date, only one HBV-encoded miRNA has been identified by deep sequencing of HBV-positive HCC tissue. The corresponding study identified and confirmed the expression of HBV-miR-3. HBV-miR-3 downregulated HBV protein and HBV replication by reducing the expression of HBcAg, a positive regulator of HBV transcription and pregenomic RNA (pgRNA), which inhibits HBV replication overall (90).

### Adenovirus

The adenovirus genome encodes two non-coding RNAs, namely VA-RNA I and VA-RNA II (91). VA RNAs are structurally like pre-miRNAs and bind and block Dicer (92, 93). However, 2–5% of VA RNAs can still be processed by Dicer in a manner similar to cellular miRNAs and can produce VA-RNA-derived miRNAs (called mivaRNAs) (92, 94). VA-RNA-I produces the two most abundant mivaRNAs (mivaRNAI-137 and mivaRNAI-138), and VA-RNA-II produces a single mivaRNA (mivaRNAII-138) (95). mivaRNAs have the potential to regulate cellular gene expression, which could be important in the adenovirus life cycle. Ectopic expression of mivaRNAI-138 in HeLa cells downregulated the expression of several genes controlling DNA repair, cell growth, apoptosis, and RNA metabolism (96). Recently, it was shown that mivaRNAII-138 enhanced Jun N-terminal kinase (JNK) signaling by downregulating CUL4, a negative regulator of the JNK signaling cascade (97). JNK signaling is crucial for viral replication, and several viruses have been reported to activate the JNK pathway upon infection (98–100). However, VA RNA-derived miRNA was dispensable for lytic replication of adenovirus in tissue culture cells. VA RNAI is a known suppressor of interferon-induced PKR enzyme, and, in the absence of PKR, the deletion of VA RNAI rescued viral late gene expression, suggesting that VA RNA-derived mivaRNAs might play an important role in inhibiting the PKR pathway to promote late gene expression (101).

### Polyomavirus

Polyomavirus are non-enveloped viruses that infect a wide range of species including humans, primates, rodents, birds, and cattle (102). Few reports have characterized miRNAs in polyomaviruses. The betapolyomaviruses BK virus (BKV), JC virus (JCV), simian virus 40 (SV40), Merkel cell polyomavirus (MKV), and simian agent 12 (SA12) have one pre-miRNA at the 3′ end that encodes two mature miRNAs. These miRNAs control the viral replication by autoregulating the viral gene expression or inhibiting the viral T antigen expression to suppress antiviral T cell response (17, 103–105).

### v-miRNAs in RNA Viruses

Most of our current understanding of v-miRNAs has been attributed to v-miRNAs that were produced by DNA viruses. The detection of v-miRNAs in RNA viruses has been controversial, with a few reports suggesting non-canonical miRNA-like small RNAs produced during RNA virus infections; however, these small RNAs lack the canonical stem-loop structure found in miRNAs, so their biogenesis and function are not well-understood (106, 107). The following reasons might explain the lack of v-miRNAs produced by RNA viruses during infection: (i) the RNA viruses consists either +/– sense or double-stranded RNA (dsRNA) and replicate in the cytoplasm of the host cell, so the RNA molecules are not accessible to the miRNA biogenesis machinery in the nuclei (108); (ii) excision of pre-miRNA from the primary transcript might result in the destruction of RNA-based viral genomes; (iii) the generated v-miRNA may target the viral genome itself, resulting in cleavage of the viral genome (109).

### Influenza Virus

The influenza virus replicates inside the host nucleus and it therefore differs from most of the other RNA viruses. As the virus replicates inside the host nucleus, it can utilize the nuclear miRNA processing factor Drosha to express v-miRNAs (108). One of the hallmarks of H5N1 influenza virus infection is a strong and rapid production of antiviral cytokines, a process known as a cytokine storm. A study using an influenza virus engineered by incorporating a primary (pri) form of cellular miRNA-124 into the genome showed the ability to produce functional miR-124 without any deleterious effects on the viral life cycle. This study suggests that RNA viruses have the ability to exploit the host's small RNA machinery to produce v-miRNAs (110). The ability to produce functional miRNAs can be harnessed to mediate the delivery of miRNAs or siRNAs using RNA virus-based vectors (111). H5N1 encodes miRNA-like small RNA named miR-HA-3p, which accentuates the production of antiviral cytokines by targeting PCBP2, a known regulator of RIG/MAVS signaling. Suppression of PCBP2 leads to high levels of cytokine production and results in high mortality (112). In another study, it was shown that the 5′ end of all the eight segments of the influenza virus encodes small viral leader RNAs (leRNAs). LeRNAs are known to play a key role in the genomic RNA encapsidation to newly generate progeny virions, suggesting an important role in the viral life cycle (113).

### Ebola Virus

Ebola virus (EBOV) is a negative-sense RNA virus that causes severe infectious disease with a high mortality rate of 83–90%. To date, few studies have identified miRNA-like small RNA encoded by EBOV (114–117). The first report was by Liang et al., who predicted that three mature miRNAs (EBOV-miR-1-5p,-3p, and miR-2-3p) are formed from two pre-miRNAs (EBOV-pre-miR-1 and EBOV-pre-miR-2) using the host cellular machinery. Further, they validated the production of mature miRNA by transfecting the pre-forms in HEK293T and showed reduced production of the mature miRNAs in Dicer-deficient cells (115). Similarly, another group performed genome-wide screening and predicted the formation of seven mature miRNAs from four pre-miRNAs (EBOV-pre-miRNA-T1, -T2, -T3, and -T4) (117). The third study identified two mature miRNAs and found that one miRNA, EBOV-miR-1-5p, serves as an analog of human miR-155 (116). Overexpression of EBOV-miR-1-5p inhibits the expression of importin-alpha5 in HEK293T cells. Reduced expression of importin-alpha5 could facilitate evasion of the host immune system (116). Although these reports identify miRNAs expressed in EBOV infection, the underlying mechanism is not so well-understood. With the development of a reverse genetics system for EBOV, characterizing the roles of these miRNAs in regulating the viral life cycle and immune response could yield insights into controlling EBOV pathogenesis. One more study showed that EBOV expresses a putative miRNA-like RNA fragment, EBOV-miR-VP-3p, which is highly conserved among other strains. EBOV-miR-VP-3p was found in the exosomes and was abundantly present in the sera of individuals infected with EBOV. Interestingly, EBOV-miR-VP-3p was detectable in the serum even before the detection of viral genomic RNA, indicating that EBOV miRNAs may serve as a biomarker for early diagnosis of EBOV infection (114).

### HIV-1

Using a computational prediction tool, one study predicted that HIV-1 might encode five putative pre-miRNAs (118). Furthermore, another study showed that nef-derived miR-N367 suppresses nef expression by targeting HIV-1 nef transcript to regulate HIV-1 virulence (119). Subsequently, two independent studies found that the HIV-1 TAR element located at the 5′ end of HIV-1 encodes two microRNAs, namely miR-TAR-5p and miR-TAR-3p. TAR miRNAs target host apoptotic genes such as ERCC1 and IER3 that are involved in DNA repair to inhibit apoptosis (120–122). Another study found miR-H3 located in the active region of reverse transcriptase. miR-H3 was found to interact with HIV-1 5′LTR and enhance promoter activity, thereby increasing viral production (123). Next-generation sequencing technology has been widely used to identify and validate HIV-1 miRNAs. However, there have been some discrepancies in the miRNAs reported in different studies. Sequencing analysis of HIV-1-infected cell lines such as TZM-bl and CD8166 and primary human CD4^+^ PBMCs did not detect any HIV-1 miRNAs (124). More importantly, the HIV-1 miRNAs reported fail to satisfy the essential criteria for classification as authentic viral miRNAs. The majority of the HIV-1 miRNAs are derived from a few locations in the genome, their size is <20 nucleotides in length, and the pri-miRNA stem loop lacks the defining properties of canonical pri-miRNA stem loops (125). These outstanding questions on the functional interplay between HIV-1 miRNAs and cellular targets provide a significant opportunity to understand the viral pathogenesis better so as to develop anti-HIV-1 therapies.

## Viral miRNAs in Immune Evasion

### Targeting the Viral Gene Expression

**Figure 2:**
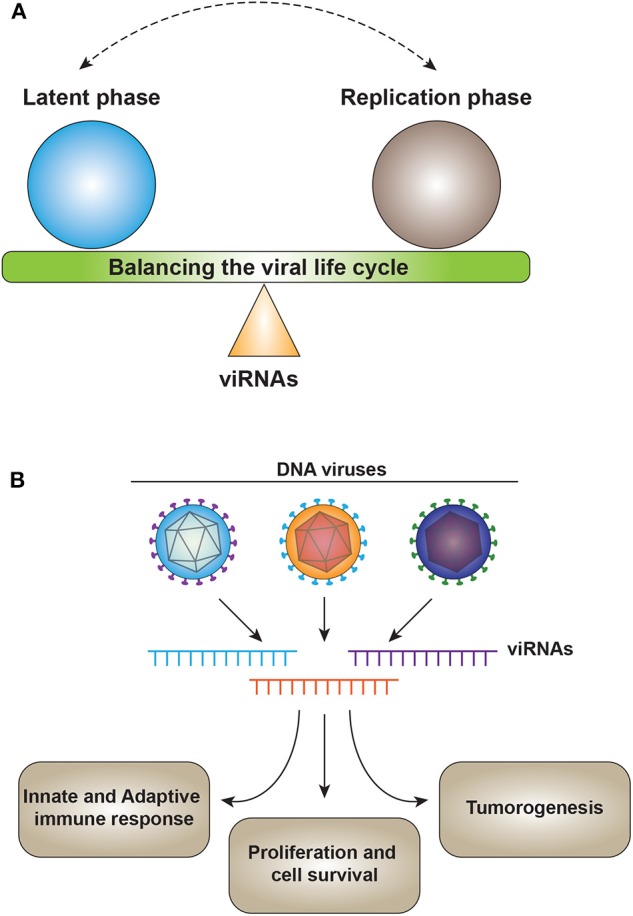
**(A)** v-miRNAs act as a fine switch between latency and the active replication phase of the viral lifecycle. **(B)** v-miRNAs control the host physiology by targeting multiple processes including immune response, cell survival, and tumorigenesis. v-miRNAs (viRNA).

One of the major functions of viral miRNAs involves targeting viral gene expression to control latency or as a switch from latency to activation (Figure 2A). SV40-encoded microRNAs regulate viral gene expression and reduce susceptibility to cytotoxic T cells (17). HSV1-induced latency is driven by LAT (Latency Associated Transcript), which encodes for non-coding RNAs such as miR-H2-3p and miR-H6. These miRNAs target viral reactivation factors ICP0 and ICP4, which are essential in controlling viral reactivation from latency of HSV-1 (79). Similarly, for HSV-2, miR-I, miR-II, and miR-III expressed by LAT reduce the expression of ICP34.5, a key viral neurovirulence factor (126). miR-I is also expressed in human sacral dorsal root ganglia of neurons latently infected with HSV-2, suggesting the role of v-miRNAs in HSV-2 latency in human neurons (127).

For HCMV, miR-UL112-1 downregulated the expression of IE72 (UL123, IE1), UL112/113, and UL120/121 by translational inhibition rather than transcript degradation (55). miR-UL112 also targets the UL114 gene, a viral uracil DNA glycosylase, with minimal effects on viral growth. In addition, human fibroblast cells (HFF) ectopically expressing miR-US25-1 and miR-US25-2 reduced viral DNA synthesis for HCMV by downregulating IE72 and pp65, one of the most abundant proteins of the virion tegument of HCMV (62). Interestingly, the antiviral effects of the ectopically expressed HCMV miRNAs miR-US25-1 and miR-US25-2 were also observed for other DNA viruses such as HSV-1 and adenovirus, suggesting that these two miRNAs might target cellular genes that are essential for virus growth or to maintain latency (62).

EBV-encoded BART miRNAs play a key role in maintaining latency and controlling the viral life cycle. BART miRNAs such as miR-BART16, miR-BART17-5p, and miR-BART1-5p target the 3′ UTR of the LMP1 gene and negatively regulate LMP1 protein expression. LMP1 is capable of inducing cell growth and proliferation, but overexpression of LMP1 can result in inhibition of growth and apoptosis. Therefore, the downregulation of LMP1 expression may favor EBV-associated cancer development by exerting tight control on LMP1 expression (128). The EBV-encoded miRNA miR-BART2 inhibits the viral transition from the latent to the lytic part of the life cycle by suppressing the viral DNA polymerase BALF5 (129). Similarly, BART20-derived v-miRNA maintains the latency phase in EBV-associated tumors by targeting two EBV immediate-early genes, BZLF1, and BRLF1 (130).

Few reports have identified v-miRNAs involved in the maintenance of KSHV latency. The KSHV-encoded miRNA miRK9 targets the viral protein RTA, a major lytic switch protein. RTA plays an important role in controlling viral reactivation from latency. v-miRNA-mediated regulation of RTA fine-tunes viral reactivation in the KSHV life cycle (119). In addition to miR-K9, miR-K12-5 can inhibit RTA expression. However, unlike miR-K9, which targets a sequence in the 3′ UTR of RTA, the 3′ UTR of RTA does not contain a favorable seed sequence for miR-K12-5, suggesting an indirect effect on RTA expression (131).

As discussed in the previous sections, the expression of v-miRNAs by RNA viruses is highly controversial. Viral miRNAs are not detected in the majority of RNA virus families, mostly due to the inaccessibility of the host machinery required for miRNA biogenesis. Small viral RNAs (svRNAs) have been identified in influenza virus infections that play a role in switching the viral polymerase from transcription toward genome replication by interacting with the polymerase machinery. However, whether svRNAs target viral or host transcripts is unknown (132). Using *in silico* tools, the HIV-1 genome was putatively shown to encode five pre-miRNAs. Based on the mature miRNA sequence deduced from the pre-miRNAs, these miRNAs were computationally predicted to target a large set of host cellular genes to establish a favorable cellular milieu for viral replication (118).

### Targeting the Host Cellular Genes

Evidence has been accumulating for v-miRNAs modulating the host immune response to enable a favorable intracellular milieu (Figure 2B). To date, studies of v-miRNAs targeting host cellular genes have been mainly focused on KSHV and EBV infections (Table 1). Host target cellular genes have been identified with the help of gene expression profiling in HEK293 cells, ectopically expressing the KSHV miRNA cluster. KSHV miRNA suppressed the expression of thrombospondin 1 (THBS1), strong tumor suppressor, and anti-angiogenic factor (159). KSHV miR-K12-1 controls cell survival and proliferation by targeting p21, a key tumor suppressor and inducer of cell cycle arrest (160). miR-K5, along with K12-9 and miR-K12-10b, targets Bcl-2-associated factor (BCLAF1), a known apoptotic factor (123). In addition, KSHV v-miRNAs reduce expression of C/EBPβ p20 (LIP), a known negative regulator of IL6 and IL10 cytokines, to regulate the cytokine signaling in infected cells (165). KSHV miR-K1 regulates the NF-κB pathway by directly targeting IκB. Suppressing IκB enhances NF-κB activity and inhibits viral lytic replication (161). KSHV miR-K12-10a suppresses the expression of TWEAKR (TNF-like weak inducer of apoptosis receptor) (169), whereas miR-K12-9 and miR-K12-5 target the TLR/Interleukin-1R signaling pathway by targeting IRAK1 and MYD88, thereby controlling inflammation (168). KSHV viral miRNAs also modulate the host gene expression to control pathogenesis. miR-K12-6 and miR-K12-11 direct the transcriptional reprogramming in latently infected cells by targeting the cellular transcription factor MAF (167). Several KSHV viral miRNAs target Retinoblastoma (Rb)-like protein 2 (Rbl-2), a negative regulator of DNA methyltransferases, to maintain latency (131). Using Ago2-based RIP-Chip in multiple B cell lines latently infected with KSHV or stably transduced to express 10 KSHV miRNAs identified genes involved in lymphocyte activation and pre-mRNA splicing such as LRRC8D and NHP2L1, respectively (139).

**Table 1:** Viral miRNAs with regulatory functions and immune evasion approaches.

**Virus**	**v-miRNA**	**Target**	**Regulation/Immune evasion**	**Model**	**References**
**DNA virus family**					
**Herpesviruses**					
HSV-1	miR-H2-3p	ICP0, ICP4	Immediate-early transactivation (latency establishment)	Male CD-1 mice, 293T and Vero cells (Δ)	(79, 80)
	miR-H3	ICP34.5	Neuro-virulence determinant	(Δ)	(79)
	miR-H4	ICP34.5	Neuro-virulence factor	(Δ)	(79)
	miR-H6	ICP0, ICP4	Immediate-early transactivation (latency establishment)	(Δ)	(79, 80)
	miR-H8	PIGT	GPI anchoring and immune evasion	BJAB cells, NK cells	(133)
HSV-2	miR-H2/H3/H4	ICP0; ICP34.5	Immediate-early transactivation (latency establishment); neuro-virulence determinant	Vero, HEK293, HeLa, and US02 cells	(126, 127)
HCMV	miR-UL-112-1	IE72	Immediate-early transactivation (latency establishment)	NHDF and U373 cells	(55, 63)
		UL114	Viral uracil DNA glycosylase	HFF cells	(62)
	miR-UL-112-1, miR-US5-1, miR-US5-2	VAMP3; RAB5C;RAB11A; SNAP23; CDC42	Host secretary pathways; control cytokine secretion; formation of VAC	NHDFs, HEK293 (VAMP3-FL-cDNA clone)	(134)
	miR-UL-112-1	MICB	Cell-mediated immunity by NK cell ligand	Cells-HFF, RKO, DU145, PC3, 1106mel, NK cells	(67)
	miR-UL-112-1, miR-US5-1	IKKα; IKKβ	Blocks NFκB signaling; partially blocks IL-1β and TNFα signaling	NHDF, THP-1, hAECs	(54)
	miR-UL-148D-1	RANTES	Activation and secretion of T cells, controls viral pathogenesis	HFF cells	(71)
		ACVR1B	Triggered secretion of IL-6, controls viral pathogenesis	HFFF2, CD34+ myeloid cells	(69)
	miR-US4-1	ERAP1, acts as biomarker	Controls MHC-I presentation to CD8^+^ T cells, indicator of IFNα treatment in hepatitis B patients	Serum (Patient)	(135)
EBV	miR-BART22	LAMP2A	Viral oncogenesis in NPC and immune evasion	HEK293T cells, biopsy samples	(136)
		NDRG1; IL12	Immune surveillance escape; T-cell differentiation, activation and recognition	B95-8, PC-3 AdAH, HEK293, and C666-1 cells; hPBMCs, B cells, T cells and LCLs	(137, 138)
	miR-BART1-5p and miR-BART5-5p	Viral LMP1	Apoptotic inducer and transforming factor, viral oncogenesis	NPC cells	(128)
	miR-BART16	LMP1	Apoptotic inducer and transforming factor, viral oncogenesis	NPC cells	(128)
		TOMM2; CBP	Mitochondrial membrane protein; immunomodulation	B cells (DG75-eGFP and DG75-10/12)	(139)
	miR-BART17-5p	LMP1	Apoptotic inducer and transforming factor, viral oncogenesis	NPC cells	(128)
		TAP2	Peptide transportation	hPBMCs, B cells, T cells, and LCLs	(138)
	miR-BART1	IL12B; IFI30	T-cell differentiation, activation and recognition; antigen processing	hPBMCs, B cells, T cells, and LCLs	(138)
	miR-BART2/BART2-5p (Viral)	BALF5	DNA polymerization and controls viral replication	BL41 cells	(129)
		MICB; LGMN and CTSB; IL12B	Cell-mediated immunity by NK cell ligand; antigen processing; T-cell differentiation, activation, and recognition	293T, RKO, HeLa, 721.221 and BCBL1 cells; NK cells; hPBMCs, B cells, T cells, and LCLs	(138, 140–142)
	miR-BART3-3p	IPO7	Nuclear import protein; T-cell activation and immune cell tolerance	B cells (DG75-eGFP and DG75-10/12)	(139)
	miR-BART5	PUMA	Apoptotic inducer and proapoptotic factor	hNPC and EBV-GC cells	(143)
	miR-BART5-3p	TP53	Cell cycle progression by inhibiting p53, inhibition of apoptosis	EBV-gastric cancer cell lines	(144)
	miR-BART8	IFN-γ	Immunomodulation	YT, NK92, and Jurkat cells	(145)
	miR-BART10-3p	IL12B	T-cell differentiation, activation, and recognition	hPBMCs, B cells, T cells, and LCLs	(138)
	miR-BART11-5p	EBF1	B-cell differentiation	B cells, LCLs, HEK293T	(146)
	miR-BART15	NLRP3; BRUCE	Inflammasome production; apoptosis	Macrophages; AGS, SUN-719 cells	(147, 148)
	miR-BART16	CBP	Immunomodulation	EBV^+^BL and SUN-719 cells	(149)
	miR-BART17	TAP2	Peptide transportation	hPBMCs, B cells, T cells, and LCLs	(138)
	miR-BART18-5p	MAP3K2	Lytic reactivation	B cells	(150)
	miR-BART20-5p	BRLF1 and BZLF1	Latency establishment	AGS, SUN-719, and YCCEL1	(130)
		BAD; IFN-γ	Apoptosis; immunomodulation	AGS, SUN-719, YCCEL1, DG75, and B cells; YT, NK92, and Jurkat cells	(145, 151)
	miR-BHRF1-2	PRDM1; CTSB; IL12B	B-cell terminal differentiation; antigen processing; T-cell differentiation, activation, and recognition	LCLs, JY25, CCL156/159, TIB190, BL cells; hPBMCs, B cells, T cells and LCLs	(138, 152)
	miR-BHRF1-3	CXCL11; TAP2	Chemokine and T-cell attractant; peptide transportation	BL-5/8, EBV^+^BL, BC-1, JCS-1, and PEL cells; hPBMCs, B cells, T cells, and LCLs	(138, 153)
	miR-BHRF1, miR-BHRF2, miR-BHRF3	BSAP1/pax5, RFX1, YY1, MIBP1, CREB, ATF1	Cellular transactivator: regulate expression of Wp/Cp promoter; viral infection persistence	B cells	(154–156)
KSHV	miR-K12-9^*^, miR-K12-5p, miR-K9^*^/miR-K5, miR-K12-7-5p	RTA	Controls viral replication and transcriptional activator (latency establishment)	HFF, BCBL-1, SLK, HEK293; DG75 and BCBL-1 cells; HEK293-Bac36 cells	(157, 158)
	miR-Cluster	THBS1 EXOC6 ZNF684 CDK5RAP1	Inhibition of angiogenesis; SEC15 gene: Zinc figure protein; regulation of neural differentiation	BCBL-1, HEK293; B cells (DG75-eGFP and DG75-10/12)	(139, 159)
	miR-K1, miR-K12-1	P21; IκBα	Inhibition of cell cycle; inhibits NFκB signaling	U2OS, BL40, HEK293T, BC-3; PEL-BCP-1 cells	(160–162)
	miR-K12-1, miR-K12-3p, miR-K12-6-3p	THBS1	Cell cycle regulation and tumor suppression	BCBL-1, HEK293	(159)
	miR-K12-1, miR-K12-3	CASP3	Inhibition of apoptosis	HEK293 and DG75 cells	(163)
	miR-K12-3	NFIB	Latency establishment	BC-3 cells	(164)
		LRRC8D; NHP2L1	Activation of immune cells; U4 snRNA nuclear binding protein	B cells (DG75-eGFP and DG75-10/12)	(139)
	miR-K12-3, miR-K12-7	C/EBPβ (LIP)	Transcriptional activator	BCBL-1, MM6, and RAW cells	(165)
	miR-K12-4	IκBα	Inhibits NFκB signaling	PEL-BCP-1 cells	(162)
	miR-K12-4-3p	GEMIN8; CASP3	Splicing factor; inhibition of apoptosis	B cells (DG75-eGFP and DG75-10/12); HEK293 and DG75 cells	(139, 163)
	miR-K12-4-5p	Rbl-2	Rb-like protein	DMVECs, HEK293T	(131)
	miR-K12-5	BCLAF1; Rbl-2	Proapoptotic factor and promotes lytic reactivation; Rb-like protein	HUVEC, BCBL-1, BJAB-B cells; DMVECs, HEK293T	(131, 166)
	miR-K12-6	MAF	Transcription factor	LECs, BECs	(167)
	miR-K12-7	MICB; NFIB	Cell-mediated immunity by NK cell ligand; latency establishment	293T, RKO, HeLa, 721.221, and BCBL1 cells, and NK cells; BC-3 cells	(140, 164)
	miR-K12-9	IRAK1 and MyD88; BCLAF1	Immune evasion; proapoptotic factor and promotes lytic reactivation	HEK293, SLK, HUVEC, BCBL-1, and BJAB-B cells	(166, 168)
	miR-K12-10a/b	TWEAKR; BCLAF1	Regulates apoptosis and inflammation; proapoptotic factor and promotes lytic reactivation	HUVEC, SLK+K cells; BCBL-1, BJAB-B cells	(166, 169)
	miR-K12-11 ^*^^*^ identical seed sequence to that of cellular miR-155	BACH1; C/EBPβ; MAF; IκBα; THSB1; NFIB	Transcriptional suppressor; splenic B-cell expansion and induces lymphomagenesis; transcription factor; inhibits NFκB signaling; cell cycle regulation and tumor suppression; latency establishment	BCP-1, BC-1, VG-1, JSC-1, RAJI, BCBL-1 BJAB; hCB cells, NOD/LtSz-scid IL2Rγ^null^ mice; LECs, BECs; PEL-BCP-1 cells; BC-3 cells	(162, 164, 167, 170–173)
**Polyomaviruses**					
SV40	miR-M1 (5p and 3p)	T Antigens (Large)	Regulation of early viral genes; transforming factor	TC-7/Db cells	(17)
SA12	miR-S1	T Antigens (Large)	Regulation of early viral genes	BSC40 cells	(105)
BKV	miR-B1	T Antigens (Large)	Regulation of early viral genes	U87 and Vero cells	(104)
JCV	miR-J1 (5p and 3p)	T Antigens (Large)	Regulation of early viral genes; transforming factor	U87 and Vero cells	(104)
MCV	miR-S1	T Antigens (Large)	Regulation of early viral genes	HEK293T cells and MCC350 plasmid	(103)
**Hepadnavirus**					
HBV	HBV-miR-3	3.4 kb viral transcript	Regulate HBc protein and pgRNA levels to alter viral replication	Huh7 and HepG2.2.15 cells	(90)
**RNA virus family**					
**Orthomyxovirus**					
Influenza	Engineered host miR-124 in intronic region of the virus	miR-124 targets	Regulate cellular functions of miR-124	HEK293, MDCK, CAD, murine fibroblast	(110)
	miR-HA-3p	PCBP2	Regulates cytokine production and viral infection	*in-vivo* mouse model	(112)
**Filovirus**					
EBoV	miR-1-3p, miR-1-5p, and miR-T3-3p	c-MET, Activin, and KPNA1 (by miR-155 ortholog)	Cellular signaling pathways, immune response dysregulation	Balb/c mice, NHP, and patients	(174)
**Flaviviruses**					
WNV	KUN-miR-1	GATA4	Regulates viral replication	Mosquito cells and bioinformatic approaches	(175)
DENV	DENV-vsRNA-5	NS-1	Autoregulation of viral replication	Mosquito cells and deep-sequencing approach	(176)
ZIKA	Putative 47 v-miRNAs	Multiple cellular targets	Immune surveillance and other biological pathways	Bioinformatic approaches	(177)
**Picornavirus**					
HAV	hav-miR-N1-3p	MAVS (*in-silico* predicted target)	Cellular antiviral pathways	KMB17 and HEK293T cells	(178)
**Retrovirus**					
HIV-I	miR-N367	Nef	Persistence viral infection	Balb/c and C3H/Hej mice; Jurkat T and MT-4T cells	(119)
	miR-TAR-5p and miR-TAR-3p	Multiple genes involved in apoptosis and cell survival	Apoptosis and viral propagation	Jurkat and J-LAT cells	(179)
	miR-H3	HIV-1 5′LTR	Enhances promoter activity and viral infection	Sup-T1, HEK293T, TZM-bl, and hPBMCs	(123)

The same study identified Epstein-Barr virus (EBV) miRNAs targeting host cellular genes in EBV-positive B cell lines. EBV miRNAs were shown to regulate cellular transport processes by targeting key genes such as TOMM22 and IPO7 (139). EBV miR-BART5 controlled proliferation and established latent infection by targeting PUMA. PUMA is known to modulate apoptosis by p53, so by suppressing PUMA, EBV miRNAs alter the susceptibility to apoptotic agents and improve host cell survival (143). In EBV-associated non-Hodgkin's lymphomas, miR-BHRF1-3 targets T cell-attracting chemokine CXCL11. Sequestering miR-BHRF1-3 by antisense oligos reversed the suppression of CXCL11 in primary cultures derived from patients with EBV-positive Burkitt's lymphoma (153).

HCMV miRNAs have been shown to target host genes involved in the antiviral immune response. miR-UL112 blocks the natural killer (NK) cell-mediated recognition of virus-infected cells by inhibiting the expression of MICB, a stress-induced ligand essential for NK-cell activity. Suppression of MICB results in decreased binding to the NKG2D receptor, thereby leading to decreased killing of virally infected cells by NK cells (67). Interestingly, MICB expression is suppressed by KSHV miR-K12-7 and EBV miR-BART2 by binding to different sites in the 3′ UTR, highlighting a common strategy for immune evasion commonly used by multiple DNA viruses (140). Additionally, HCMV miR-US4-1 targets the endoplasmic reticulum-resident aminopeptidase ERAP1, which is required for MHC class I antigen presentation on CD8 T cells, resulting in less clearance of infected cells by HCMV-specific cytotoxic T cells (66).

### Therapeutic Potential of Viral miRNAs

Combating viral diseases and virus-associated cancers is an ongoing health-related challenge present globally. Recent studies have focused on identifying miRNAs as targets for treating several diseases, including viral diseases. Antagomirs designed to sequester host miR-122 involved in HCV infection entered the phase II of human clinical trial and show promising effects against the infection (180, 181). Using similar approaches, v-miRNAs could also be targeted by using inhibitor or sponge-based antagomirs in DNA virus infections. A few reports support the notion of developing antagomirs against v-miRNAs rather than cellular miRNAs, as this could reduce the possibility of side-effects or off-target effects in human subjects and might solve the problem of non-toxic/site-specific, targeted delivery (182). In mouse cytomegalovirus (MCMV) infection, mice receiving antagomirs against MCMV v-miRNAs had reduced occurrence of MCMV upon challenge. In another study, gold nanoparticles containing anti-EBV-miR-BART7-3p were shown to therapeutically deliver anti-miRNAs against EBV-miR-BART7-3p, inhibiting the tumorigenicity of EBV-positive cells in mice (20, 183).

Similarly, using EBV promoters such as EBER2 promoter was reported to effectively express miRNA sponge to silence specific genes in EBV-infected cells and might be useful in the targeting of EBV-positive NPC cells (184). In addition, v-miRNAs were also considered as biomarkers in many virally infected diseases. miR-VP-3p was found to be present in the sera of EBOV-infected patients but not in healthy controls, and this v-miRNA was detectable in the serum prior to the detection of viral genomic RNA, indicating that miR-VP-3p may serve as a biomarker for early diagnosis of EBOV (114). HCMV-encoded miR-US4-1 serves as a biomarker for IFNα treatment potency in the serum of hepatitis B patients (135). Moreover, certain v-miRNA adapters (HSUR2) were discovered that recruit miRNA to target transcripts through alternative base-pairing. Inhibitors against these supportive v-miRNAs adaptors could be considered as an alternative therapeutic candidate (185). Finally, critical design and validation of miRNA-based studies in cell lines and animal models can help identify novel therapeutic candidates for treatments in the future.

## Conclusion

Although the biogenesis, mechanism and function of virally encoded miRNAs are not well-characterized, substantial progress has been made in the last few years. With the emergence of high-throughput sequencing technologies and computational analysis tools, the number of newly discovered v-miRNAs is increasing. Multiple lines of evidence have strengthened the “classical” hypothesis of v-miRNAs solely originating from DNA viruses; however, some non-canonical miRNA-like RNA fragments have been detected during RNA virus infections. While the major functions of v-miRNAs across divergent virus families have been broadly attributed to immune evasion, autoregulation of the viral life cycle and tumorigenesis, there is still a broad gap in annotating the exact molecular determinants underlying these functions. The holy grail of the functional importance of v-miRNAs warrants more investigation to provide therapeutically amenable leads for targeting infectious diseases in the future.

## Author Contributions

All authors listed have made a substantial, direct and intellectual contribution to the work, and approved it for publication.

### Conflict of Interest

The authors declare that the research was conducted in the absence of any commercial or financial relationships that could be construed as a potential conflict of interest.
